# Engineering probiotic *Escherichia coli* for inflammation-responsive indoleacetic acid production using RiboJ-enhanced genetic circuits

**DOI:** 10.1186/s13036-025-00479-y

**Published:** 2025-01-21

**Authors:** Seung-Gyun Woo, Seong Keun Kim, Seung-Goo Lee, Dae-Hee Lee

**Affiliations:** 1 Synthetic Biology Research Center and the K-Biofoundry, Korea Research Institute of Bioscience and Biotechnology (KRIBB), Daejeon, 34141 Republic of Korea; 2 Department of Molecular Biosciences, University of Texas at Austin, Austin, TX 78712 USA; 3 Department of Biosystems and Bioengineering, KRIBB School of Biotechnology, University of Science and Technology (UST), Daejeon, 34113 Republic of Korea; 4 Graduate School of Engineering Biology, Korea Advanced Institute of Science and Technology (KAIST), Daejeon, 34141 Republic of Korea; 5 Department of Integrative Biotechnology, College of Biotechnology and Bioengineering, Sungkyunkwan University, Suwon-si, 16419 Gyeonggi-do Republic of Korea

**Keywords:** *Escherichia coli* Nissle 1917, Inflammatory bowel disease, Indoleacetic acid, RiboJ insulator, Genetic circuit

## Abstract

**Background:**

As our understanding of gut microbiota’s metabolic impacts on health grows, the interest in engineered probiotics has intensified. This study aimed to engineer the probiotic *Escherichia coli* Nissle 1917 (EcN) to produce indoleacetic acid (IAA) in response to gut inflammatory biomarkers thiosulfate and nitrate.

**Results:**

Genetic circuits were developed to initiate IAA synthesis upon detecting inflammatory signals, optimizing a heterologous IAA biosynthetic pathway, and incorporating a RiboJ insulator to enhance IAA production. The engineered EcN strains demonstrated increased IAA production in the presence of thiosulfate and nitrate. An IAA-responsive genetic circuit using the IacR transcription factor from *Pseudomonas putida* 1290 was also developed for real-time IAA monitoring.

**Conclusions:**

Given IAA’s role in reducing gastrointestinal inflammation, further refinement of this strain could lead to effective, in situ IAA-based therapies. This proof-of-concept advances the field of live biotherapeutic products and offers a promising approach for targeted therapy in inflammatory bowel diseases.

**Supplementary Information:**

The online version contains supplementary material available at 10.1186/s13036-025-00479-y.

## Background

The intricate interplay between host organisms and their gut microbiota is pivotal in maintaining health and influencing various diseases [1]. Disruptions in the gut microbiome are linked to a range of conditions, including metabolic syndrome [2], immune system malfunctions [3], obesity [4], inflammatory bowel disease (IBD) [5], and even neuropsychiatric disorders [6]. Gut bacteria produce metabolites that play crucial roles in these host-microbiota interactions, significantly affecting health and disease dynamics.

This intricate connection between microbial metabolites and host health underpins the development of live biotherapeutic products (LBPs). LBPs are a novel class of probiotics engineered to perform therapeutic functions directly within the host [7]. Advances in systems and synthetic biology have enabled the design of these probiotics to produce specific therapeutic molecules and perform precise functions tailored to the host environment [8]. These microorganisms are equipped with genetic circuits that detect environmental signals (biomarkers), process these signals, and initiate appropriate responses. Each component is modularized into bioparts, allowing flexible adjustments in the ‘Sensing-and-Responding’ functionality of these cellular systems. This genetic architecture enables LBPs to undertake diverse activities based on predefined environmental inputs and desired therapeutic outputs, including precise localization [9], biocontainment [10], controlled drug delivery [11], and regulated colonization [12].

Secondary metabolites, predominantly produced by the gut microbiota, are crucial in this context [13, 14]. The dynamics within the microbial community significantly influence the production of these beneficial compounds [15–17]. Strategies in LBP development often aim to augment the production of these metabolites, particularly when natural microbial production is reduced by antibiotics or disease-associated alterations in the microbiome. For example, the breakdown of dietary proteins releases tryptophan, which is then converted by gut microbiota such as *Bacteroides* species and *Clostridium bartlettii* into catabolites like indoleacetic acid (IAA) [18]. IAA, acting as an aryl hydrocarbon receptor (AHR) ligand [19], is pivotal in immune modulation, regulating both innate and adaptive immune systems [20–22] and promoting protective and anti-inflammatory responses in the gut mediated by interleukin (IL)-22 [23] and innate lymphoid cells [24]. Additionally, IAA has been shown to enhance the efficacy of chemotherapy for pancreatic ductal adenocarcinoma (PDAC), with evidence demonstrating that dietary modulation or supplementation to increase IAA levels improves treatment outcomes, particularly in chemotherapy-resistant PDAC patients [25]. Given IAA’s role and safety profile within the human intestine, it presents a viable therapeutic avenue, especially for IBD such as ulcerative colitis (UC) and Crohn’s disease (CD), which profoundly affect quality of life. Recent research underscores the connection between IBD and alterations in gut microbiota signaling [13] and tryptophan metabolism [26], highlighting the potential of AHR-targeted therapies in IBD management.

Our research advances this potential by engineering the probiotic strain *Escherichia coli* Nissle 1917 (EcN) to enhance IAA production in response to specific inflammation-related biomarkers—thiosulfate (S_2_O_3_^2−^) [27] and nitrate (NO_3_^−^) [28]. This engineering effort began with the optimization of an IAA biosynthetic pathway in the EcN strain, followed by the integration of the RiboJ insulator to boost IAA synthesis. Subsequently, we developed EcN strains that not only detect but also respond robustly to elevated levels of these biomarkers, signifying inflammation. Moreover, we introduced an IAA-responsive genetic circuit designed for real-time IAA monitoring, thereby facilitating both intracellular and environmental assessments of IAA levels.

## Methods

### Strains and culture conditions

The *E. coli* strains used in this study are detailed in Table S1. *E. coli* DH5α served as the host strain for plasmid construction, while the EcN strain was employed for characterizing IAA biosynthesis and the IAA-biosensor. All *E. coli* strains were typically grown at 37 °C in lysogeny broth (LB) medium (10 g/L tryptone, 5 g/L yeast extract, and 10 g/L NaCl) with shaking at 200 rpm. For IAA biosynthesis experiments, an IAA production medium was used, which is LB medium fortified with varying concentrations of L-tryptophan and 5 g/L KH_2_PO_4_ at pH 7.0. Additional supplements to the medium, such as ampicillin, sodium nitrate, potassium thiosulfate, IAA, and Isopropyl-β-D-thiogalactopyranoside (IPTG), were added as necessary. All analytical standard materials, including L-tryptophan, indolepyruvic acid (IPyA), and IAA, were purchased from Sigma-Aldrich (Saint Louis, MO, USA). The Gibson Assembly master mix was obtained from New England Biolabs (Beverly, MA, USA).

### Plasmid construction

The complete lists of plasmids and oligonucleotide primers used in this study, as well as the DNA sequences of the *iad1*, *aspC*, *ipdC*, and *iacR* genes, are available in Tables S1, S2, and S3, respectively. All recombinant plasmids were constructed using the Gibson Assembly method as per the manufacturer’s instructions. All DNA oligonucleotides were synthesized by Macrogen (Seoul, Korea). Standard protocol was followed for PCR amplifications, employing KOD One PCR Master Mix (Toyobo). The PCR fragments were treated with DpnI and purified using the LaboPass Gel and PCR Clean-up kit (CosmoGenetech). The accuracy of the constructed plasmid sequences was confirmed through Sanger sequencing at Macrogen.

The generation of the pTSN-IAA1, pTSN-IAA2, and pTSN-IAA3 plasmids involved producing and assembling three distinct fragments for each plasmid. For pTSN-IAA1, F1, containing the *aspC* gene, was amplified from genomic DNA of *E. coli* MG1655 using aspC-IF and aspC-IR primers. F2, with codon-optimized *ipdC* and *iad1* genes, was derived from synthesized DNA using aspC-VF and iad1-IR primers. F3, comprising the backbone of the pTrc99A plasmid, was amplified with iad1-VF and aspC-VR primers. These were assembled via Gibson Assembly to form pTSN-IAA1. To create pTSN-IAA2, F1, F2, and F3 were produced from pTSN-IAA1 plasmids. F1 was obtained through PCR with iad1-IF and iad1-IR, F2 with aspC(B0034)-IF and ipdC-IR, and F3 with rrnBT-VF and lacO-VR primers. These fragments were then assembled to generate pTSN-IAA2. For pTSN-IAA3, F1, containing the RiboJ insulator, was prepared from synthesized DNA using RiboJ-IF and RiboJ-IR primers. F2 and F3, derived from the pTSN-IAA2 plasmid, were prepared with RiboJ(iad1)-VF and ipdC-IR for F2, and rrnBT-VF and RiboJ(lacO)-VR for F3. These fragments were then assembled to create the pTSN-IAA3 plasmid.

The pPhsA-IAA4, pPhsA-IAA5, pPhsA-IAA6, pPhsA-IAA7, pYeaR-IAA1, and pYeaR-IAA2 plasmids were all synthesized using a two-fragment assembly approach. For pPhsA-IAA4 and pPhsA-IAA5, the F1 fragment was PCR-prepared from the pTSN-IAA2 plasmid using iad1(B0034)-IF and ipdC-IR1 primers. The F2 fragment for pPhsA-IAA4 came from the pSEVA131-J23114-TPG plasmid, while for pPhsA-IAA5, it was from the pSEVA131-J23114-T(D57A)PG plasmid, both using ipdC-VF and ThsS(B0034)-VR primers. These fragments were assembled via the Gibson Assembly method to create the respective plasmids. In a similar fashion, pPhsA-IAA6 and pPhsA-IAA7 were generated, with F1 from the pTSN-IAA3 plasmid using RiboJ(PphsA)-IF and ipdC-IR1 primers. F2 for pPhsA-IAA6 was from the pSEVA131-J23114-TPG plasmid, and for pPhsA-IAA7, from the pSEVA131-J23114-T(D57A)PG plasmid, both using ipdC-VF and RiboJ(PphsA)-VR primers.

Similarly, for pYeaR-IAA1 and pYeaR-IAA2, F1 was prepared from the pTSN-IAA3 plasmid using RiboJ(PyeaR)-IF and ipdC-IR1 primers. F2 for pYeaR-IAA1 was derived from the pSEVA131-J23113-NPG plasmid, while for pYeaR-IAA2, it came from the pSEVA131-J23113-N(H399A)PG plasmid, both using ipdC-VF and RiboJ(PyeaR)-VR primers. These fragments were then assembled via the Gibson Assembly method to create the respective pYeaR plasmids.

The inducible promoters (pPhsA and pYeaR) used in the inflammation-responsive genetic circuits were previously characterized for their steady-state transfer functions and dynamic response times [28]. This analysis provided insights into promoter behavior under various conditions, informing the selection and optimization of circuits for timely and robust activation of IAA production in response to specific biomarker concentrations.

To generate the pIacA-sfGFP plasmid, two fragments, F1 and F2, were prepared. The F1 fragment, which includes the codon-optimized *IacR* regulator, constitutive P_*J23114*_ promoter, and native P_*iacA*_ promoter, was amplified from synthesized DNA using the iacR-IF and PiacA(B0034)-IR primers. The F2 fragment, forming the backbone of the pIacA-sfGFP plasmid, was amplified from the pSEVA131-J23114-TPG plasmid using PiacA(B0034)-VF and iacR-VR primers. These fragments were then assembled using the Gibson Assembly method to create the pIacA-sfGFP plasmid.

### IAA production

To evaluate IAA production, individual colonies were picked from LB plates supplemented with 100 µg/mL ampicillin and inoculated into 3 mL of LB liquid medium with 100 µg/mL ampicillin. They were cultured at 37 °C with shaking at 200 rpm overnight to prepare the seed culture. Subsequently, 50 µL of this seed culture was added to 5 mL of IAA production medium, also supplemented with 100 µg/mL ampicillin, and the cells were cultivated for an additional 24 h for IAA biosynthesis. Typically, 2 g/L L-tryptophan was added to the IAA production medium. In the IPTG-inducible system, IPTG was added to the medium at final concentrations of 0, 0.05, 0.1, and 0.2 mM when the optical density at 600 nm (OD_600_) of the cultured cells reached 0.6 − 0.8. For the inflammatory biomarker-inducible system, sodium nitrate or potassium thiosulfate was added at final concentrations of 0, 0.04, 0.2, and 1 mM at the beginning of the main culture. To explore the correlation between L-tryptophan concentration and IAA biosynthesis, final concentrations of 0, 0.5, 1, and 2 g/L L-tryptophan were used. After completing IAA production, 100 µL of the cell culture was diluted 1:10 with PBS buffer to a total volume of 1 mL. The OD_600_ was then measured using a UV-VIS spectrophotometer (Ultrospec 8000, GE Healthcare, Uppsala, Sweden) to assess cell growth.

### Quantitative analysis of IAA

For quantitative determination, high-performance liquid chromatography (HPLC) analysis was performed to determine the concentrations of IAA produced, following a previously described method with some modifications [29]. Briefly, 1 mL of cell culture was collected and adjusted to pH 2.5 with 10% H_3_PO_4_ and extracted with an equal volume of ethyl acetate using vigorous vortexing for 5 min. The sample extract was then centrifuged at 15,000 xg for 10 min at 4 °C to separate the phase. Next, 1 mL of the top layer was transferred into a fresh microcentrifuge tube and evaporated to dryness. The dried samples were redissolved in 1 mL of methanol and filtered through a 0.45-µm syringe filter. HPLC analysis was conducted on an Agilent Technologies 1200 series system (Santa Clara, CA), equipped with a refractive index detector (RID) and a Zorbax Eclipse XDB-C18 column (150 mm x 4.6 mm, Agilent). The oven temperature was maintained at 35 °C. Quantification of L-tryptophan, IPyA, and IAA was performed at a flow rate of 1 mL/min using a mobile phase of 30:70 (v: v) acetonitrile and 1% acetic acid in HPLC-grade water. The detection wavelength was set at 280 nm. The presence of L-tryptophan, IPyA, and IAA was confirmed by retention time comparison. A standard curve was created using six different concentrations of HPLC-grade authentic standards of L-tryptophan, IPyA, and IAA. Quantification was conducted by correlating the peak areas of the sample extracts with this standard curve.

### Characterization of IAA biosensors

EcN cells harboring the placA-sfGFP plasmid underwent initial overnight growth at 37 °C with shaking at 200 rpm in 3 mL of LB media supplemented with 100 µg/mL ampicillin. This overnight culture was subsequently diluted 100-fold in fresh LB media, supplemented with 100 µg/mL ampicillin and different IAA concentrations ranging from 0 to 1 mM. Then, 200 µL of this culture was aliquoted into each well of a 96-well CELLSTAR^®^ microplate (Greiner Bio-One) with black walls and clear bottoms. For time course experiments, the microplate was incubated for 24 h at 37 °C in a Tecan Infinite 200 PRO microplate reader (Männedorf, Switzerland) to monitor cell growth and fluorescence signal. Cell growth was measured at an OD_600_, and fluorescence intensities were recorded with excitation and emission wavelengths set at 488 nm and 515 nm, respectively.

## Results and discussion

### Optimization of the IAA biosynthetic pathway

Tryptophan, an essential amino acid derived from the diet, is metabolized by gut microbiota into various indole derivatives, including IAA, a microbiota-derived ligand of the aryl hydrocarbon receptor (AHR). IAA is known to enhance the production of IL-22, which is critical for maintaining intestinal health and mitigating inflammation [26, 30]. AHR signaling via IL-22 plays a pivotal role in suppressing inflammation and preventing colitis in the gastrointestinal tract [31, 32]. Notably, reduced fecal IAA concentrations and downregulated AHR expression have been observed in patients with IBD, underscoring a potential therapeutic target [26, 32].

**Fig. 1:**
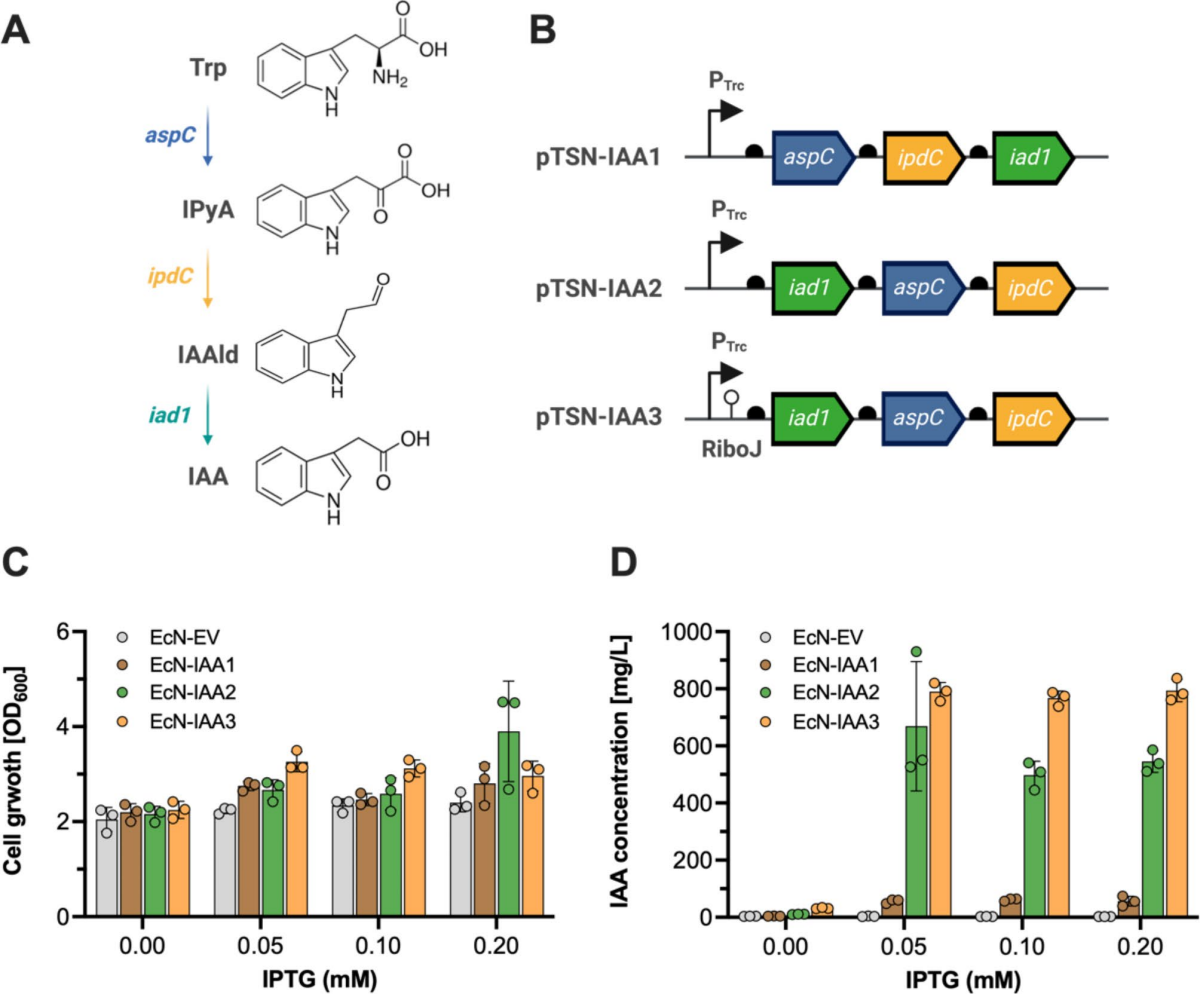
Biosynthesis of IAA in*E. coli*Nissle 1917 strain. (**A**) Schematic representation of the heterologous IPyA pathway for IAA production from tryptophan (Trp) in the EcN strain. The IPyA pathway consists of three genes: *aspC*, *ipdC*, and *iad1*. The *aspC* gene encodes tryptophan aminotransferase, the *ipdC* gene encodes IPyA decarboxylase, and the *iad1* gene encodes indoleacetaldehyde (IAAId) dehydrogenase. (**B**) Engineering of the IAA biosynthesis plasmids for enhanced production of IAA. The composition of the IPTG-inducible IAA biosynthesis plasmids (pTSN-IAA1, pTSN-IAA2, and pTSN-IAA3) is depicted. The EcN strains were transformed with plasmids pTSN-IAA1, pTSN-IAA2, and pTSN-IAA3 to create the EcN-IAA1, EcN-IAA2, and EcN-IAA3 strains, respectively. (**C**) Cell growth (OD_600_) of the engineered EcN strains (EcN-EV, EcN-IAA1, EcN-IAA2, and EcN-IAA3). EV represents the pTrc99A empty vector. (**D**) IAA titers (mg/L) of the engineered EcN strains. The engineered EcN strains were cultivated in IAA production medium with 2 g/L tryptophan and induced with different concentrations of IPTG at an OD_600_ of 0.6–0.8 for 24 h at 37 °C. After that, cell growth and IAA concentrations were measured using a UV-VIS spectrophotometer and HPLC analysis, respectively. Data represent the mean and standard deviation (SD) from three biological replicates

In this study, we utilized the probiotic EcN strain as a chassis for engineering a controlled IAA production system. Leveraging its genetic tractability, beneficial properties [33, 34], and the previous studies of IAA production [29, 35], we constructed and optimized a metabolic pathway specifically for IAA biosynthesis using the IPyA pathway (Fig. 1A). This pathway involves three key enzymes: tryptophan aminotransferase (encoded by *aspC* from *E. coli*), IPyA decarboxylase (encoded by *ipdC* from *Enterobacter cloacae*), and indoleacetaldehyde dehydrogenase (encoded by *iad1* from *Ustilago maydis*).

The engineered strains EcN-IAA1, EcN-IAA2, and EcN-IAA3 were developed by transforming EcN with plasmids pTSN-IAA1, pTSN-IAA2, and pTSN-IAA3, respectively. These plasmids contain an IPTG-inducible Trc promoter to regulate the expression of three enzymes in IPyA pathway and differ primarily in the arrangement and regulatory elements of the IAA biosynthesis genes (Fig. 1B). Briefly, pTSN-IAA1 arranges the genes in the order *aspC*, *ipdC*, and *iad1* under the Trc promoter, whereas pTSN-IAA2 reorders them to *iad1*, *aspC*, and *ipdC* under the same promoter. Since Iad1 is the key enzyme in IAA biosynthesis in *Ustilago maydis* [36], the *iad1* gene was placed at the beginning of the pTSN-IAA2 construct to enhance its expression level. pTSN-IAA3 retains the gene order from pTSN-IAA2 but introduces the RiboJ insulator before *iad1* to improve gene expression efficiency. RiboJ is a synthetic ribozyme (insulator) used to regulate gene expression. RiboJ has been used to reduce the interactions between genetic elements, thereby enabling more precise control and predictable control of desired gene expression patterns within genetic circuits [37]. Additionally, the insulator RiboJ, a self-cleaving synthetic ribozyme, has been reported to significantly boost protein and RNA expression levels across various promoter strengths within genetic circuits in *E. coli* [38].

In this study, we adopted a polycistronic expression strategy in the pTSN-IAA1, pTSN-IAA2, and pTSN-IAA3 constructs to simplify plasmid design, reduce size, and enhance the efficiency of cellular resource utilization. This approach enables the simultaneous expression of *iad1*, *aspC*, and *ipdC* from a single mRNA transcript, facilitated by the RiboJ insulator to stabilize mRNA and standardize ribosome binding. Compared to monocistronic systems that employ separate P_Trc_-inducible promoters for each gene, the polycistronic strategy addresses imbalances in transcription and translation. Such imbalances often hinder metabolic efficiency, particularly in complex pathways like IAA production. While direct comparisons were not performed, prior studies highlight the advantages of polycistronic systems in maintaining balanced enzyme expression and reducing the metabolic burden [39]. These characteristics underpin the design of pTSN-IAA3, leading to the observed improvements in IAA production.

To assess the efficiency of these engineered strains, we conducted a series of experiments measuring both cell growth and IAA production under varying IPTG concentrations (Fig. 1C and D). Cell growth, measured by OD_600_, showed that all engineered strains (EcN-IAA1, EcN-IAA2, and EcN-IAA3) maintained similar growth profiles to the control strain containing the empty vector pTrc99A (Fig. 1C). This indicates that the genetic modifications did not adversely affect the growth of the EcN strains under the tested conditions.

To confirm IAA production in the EcN-IAA1 strain, we inoculated the cells in IAA production medium with IPTG and incubated them for 24 h at 37 °C. After incubation, we extracted IAA from the cells using ethyl acetate. HPLC analysis revealed a peak with the same retention time as the IAA standard (TR = 4.09 min, Fig. S1), indicating successful IAA biosynthesis by the engineered pathway in the presence of IPTG. However, the presence of an additional peak in the HPLC analysis of the EcN-IAA1 strain extracts indicates the production of by-products or incomplete pathway efficiency, emphasizing the need for further pathway optimization. Thus, we created two additional EcN strains producing IAA: EcN-IAA2 and EcN-IAA3.

The IAA production levels varied significantly among the engineered strains. In the absence of IPTG, baseline IAA production was observable, with the highest yield noted in the EcN-IAA3 strain (EcN-IAA1, 4.14 ± 0.23 mg/L; EcN-IAA2, 10.65 ± 0.68 mg/L; EcN-IAA3, 31.83 ± 2.52 mg/L), suggesting a basal level expression from the RiboJ-enhanced setup (Fig. 1D). Upon induction with 0.2 mM IPTG, IAA production in the EcN-IAA2 and EcN-IAA3 strains increased dramatically, yielding approximately 10- (545.18 ± 38.01 mg/L) and 14-fold (793.1 ± 39.41 mg/L) increases compared to EcN-IAA1 (56.59 ± 17.27 mg/L).

The comparative analysis of IAA production across different strains showed that optimizing gene order and regulatory elements within the plasmids can significantly influence the metabolic output. The RiboJ insulator stabilizes mRNA and enhances protein expression levels in *Escherichia coli*, with increases ranging from two- to tenfold depending on promoter strength [38]. Previous studies have demonstrated that RiboJ improves mRNA stability and increases translation efficiency of downstream ribosome binding sites [40]. These findings align with our observations, where the inclusion of the RiboJ insulator significantly enhanced IAA production in the pTSN-IAA3 construct.

### Development of the thiosulfate-inducible IAA production

While IL-22 provides beneficial effects, prolonged activation of the IL-22 pathway can heighten the risk of colitis-associated cancer, particularly in individuals with long-standing IBD [41]. To address this, we developed an IAA production system in EcN that responds specifically to thiosulfate, an inflammatory biomarker. This system is designed to produce IAA only in the presence of inflammatory signals, preventing continuous IAA production in the gut.

Initially, we confirmed IPTG-inducible IAA production in EcN. Next, we developed a thiosulfate-inducible IAA production system using a thiosulfate-responsive genetic circuit. This circuit is activated by thiosulfate, a known gut inflammation biomarker [27].

When thiosulfate is present, ThsS, which is constitutively expressed, undergoes phosphorylation. This, in turn, leads to the phosphorylation of ThsR, which activates the expression of the *sfgfp* reporter gene via the PhsA promoter [27]. We implemented the iad1-aspC-ipdC or RiboJ-iad1-aspC-ipdC expression cassettes under the PhsA promoter, replacing the sfGFP reporter. Additionally, we modified the IAA production plasmid from a pMB1 origin of replication to a pBBR1 origin, which is more stable for consistent gene expression and plasmid maintenance over time, even under non-selective conditions [42]. These plasmids have proven functional in the gut of DSS-colitis mouse models [28].

**Fig. 2:**
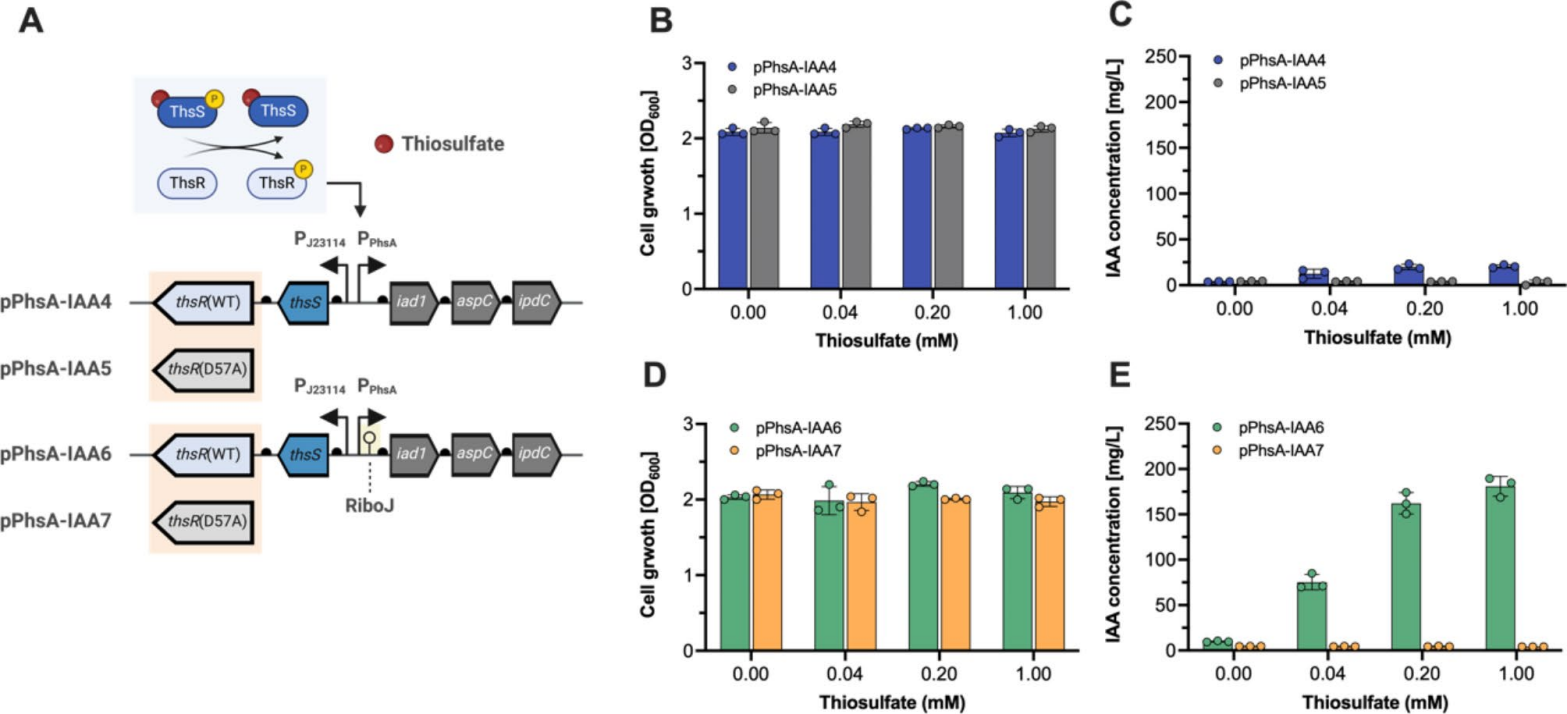
Development and characterization of the thiosulfate-inducible IAA biosynthesis. (**A**) Schematic representation of the thiosulfate-inducible IAA-producing plasmids, comprising both the thiosulfate-responsive genetic circuit and the IAA biosynthesis pathway. The ThsS-ThsR proteins of the two-component regulatory system (TCS) are constitutively expressed from the J23114 promoter. The binding of thiosulfate to ThsS triggers a phosphorylation process, creating a complex that phosphorylates ThsR. This phosphorylated ThsR subsequently activates the PphsA promoter, which in turn triggers the expression of the IAA biosynthesis pathway. The pPhsA-IAA4 and pPhsA-IAA5 plasmids contain an operon of *iad1*, *aspC*, and *ipdC* genes with the B0034 RBS for each, while pPhsA-IAA6 and pPhsA-IAA7 additionally have a RiboJ insulator before the *iad1* gene. The pPhsA-IAA5 and pPhsA-IAA7 plasmids include the ThsR(D57A) null mutant. (**B**) Cell growth (OD_600_) and (**C**) IAA titer (mg/L) for pPhsA-IAA4 and pPhsA-IAA5 in the engineered EcN strains with increasing thiosulfate concentrations (0, 0.04, 0.2, and 1 mM). (**D**) Cell growth (OD_600_) and (**E**) IAA yield (mg/L) for pPhsA-IAA6 and pPhsA-IAA7 plasmids in the engineered EcN strains in response to the increasing concentrations of thiosulfate (0, 0.04, 0.2, and 1 mM). The engineered EcN strains were cultivated in an IAA production medium with 2 g/L tryptophan and various thiosulfate levels for 24 h at 37 °C. Cell growth and IAA concentrations were measured using a UV-VIS spectrophotometer and HPLC analysis, respectively. Data represent the mean and SD from three biological replicates

Building on previous studies that showed the L547T mutation in the ThsS sensory histidine kinase enhances thiosulfate biosensor performance [28, 43], we created the pPhsA-IAA4 (ThsS(L547T)-pPhsA-iad1-aspC-ipdC) and pPhsA-IAA6 (ThsS(L547T)-pPhsA-RiboJ-iad1-aspC-ipdC) plasmids (Fig. 2A). For comparison, we generated ThsR(D57A) null mutants in each plasmid by substituting the phosphoryl-accepting aspartate residue with alanine. This modification prevents phosphorylation of the ThsR response regulator, inhibiting gene expression under the PhsA promoter, resulting in pPhsA-IAA5 (ThsS(L547T)R(D57A)-pPhsA-iad1-aspC-ipdC) and pPhsA-IAA7 (ThsS(L547T)R(D57A)-pPhsA-RiboJ-iad1-aspC-ipdC) plasmids. This setup ensured that IAA production was specifically in response to thiosulfate detection.

To evaluate if these plasmids could induce the IAA biosynthesis pathway in a thiosulfate-dose-dependent manner, we transformed EcN cells with each plasmid, generating the strains EcN-IAA4, EcN-IAA5, EcN-IAA6, and EcN-IAA7. These strains were treated with increasing thiosulfate concentrations (0 to 1 mM) for 24 h in IAA production medium. HPLC analysis assessed IAA production, and OD_600_ measured cell growth at varying thiosulfate concentrations.

Interestingly, cell growth remained consistent across all thiosulfate concentrations in the tested strains (Fig. 2B and D). However, IAA production increased with higher thiosulfate concentrations in EcN-IAA4 and EcN-IAA6 strains (Fig. 2C and E). At 1 mM thiosulfate, EcN-IAA4 produced 20.6 ± 1.66 mg/L of IAA compared to 3.97 ± 0.28 mg/L without thiosulfate, indicating a 5-fold increase. Similarly, EcN-IAA6 produced 180.96 ± 10.94 mg/L of IAA at 1 mM thiosulfate, compared to 9.87 ± 0.57 mg/L without it, showing a 43-fold increase. The EcN-IAA5 and EcN-IAA7 strains, with the ThsR(D57A) null mutation, consistently produced around 4 mg/L of IAA regardless of thiosulfate presence, confirming the specificity of the thiosulfate response.

The significant difference in IAA production between EcN-IAA4 and EcN-IAA6 strains can be attributed to the interplay of plasmid origin and the presence of RiboJ. Both strains utilize the low-to-medium-copy-number pBBR1 ori, which impacts gene dosage and, consequently, the expression levels of biosynthetic enzymes. The RiboJ insulator in EcN-IAA6 compensates for this limitation by enhancing mRNA stability and translation efficiency, resulting in a substantial increase in IAA production. This highlights the importance of integrating RiboJ in plasmids with lower replication origins, as it helps maximize pathway efficiency under constraints of reduced gene copy number.

Given that IAA can stimulate AHR-DNA response element binding in vitro with an effective concentration (EC_50_) of 0.2 to 0.5 mM, the EcN-IAA6 strain, producing IAA above this threshold, shows great promise for practical applications.

**Fig. 3:**
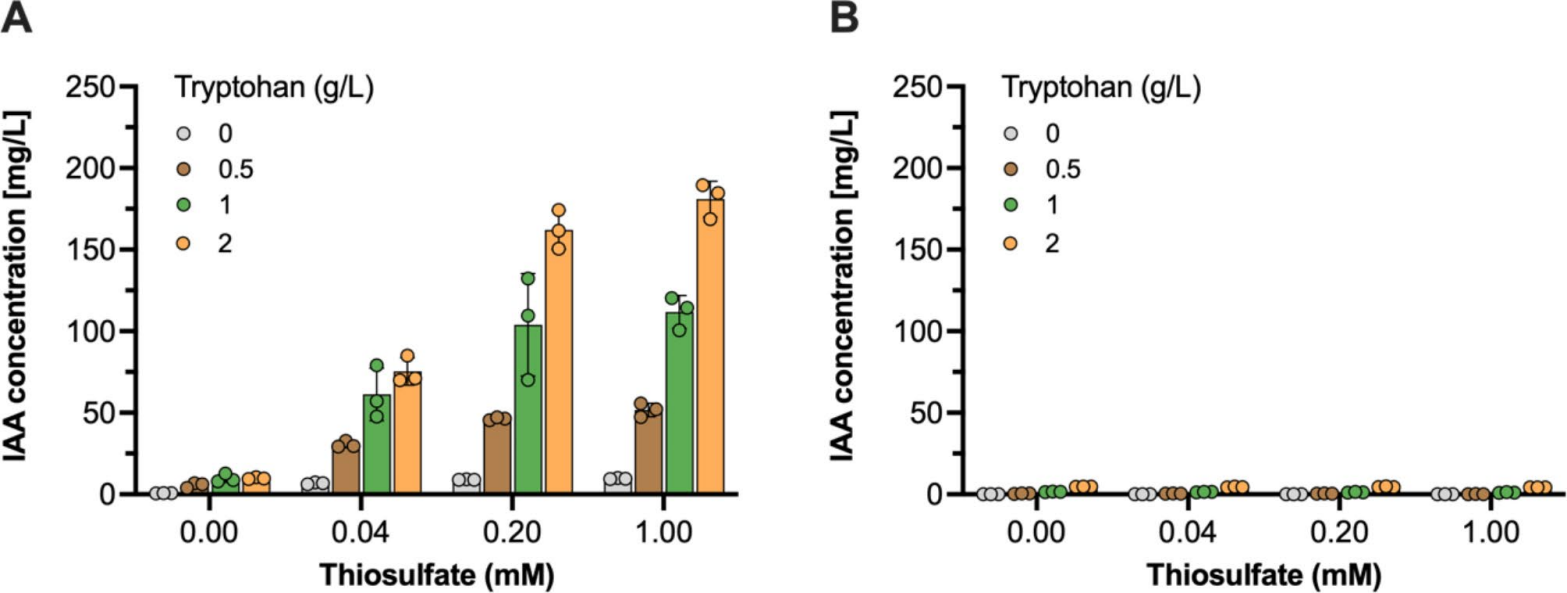
Functional characterization of the thiosulfate-inducible IAA biosynthesis. (A and B) IAA titer (mg/L) of the engineered EcN strains harboring (**A**) pPhsA-IAA6 or (**B**) pPhsA-IAA7 plasmids in response to various combinations of tryptophan and thiosulfate concentrations. The engineered EcN strains were cultivated in an IAA production medium with various combinations of tryptophan (0, 0.5, 1, and 2 g/L) and thiosulfate (0, 0.04, 0.2, and 1 mM) levels for 24 h at 37 °C. Cell growth and IAA concentrations were measured using a UV-VIS spectrophotometer and HPLC analysis, respectively. Data represent the mean and SD from three biological replicates

To further evaluate if the thiosulfate-inducible IAA production system operates in a manner dependent on both tryptophan and thiosulfate doses, we treated the EcN-IAA6 and EcN-IAA7 strains with varying concentrations of tryptophan and thiosulfate (0 to 1 mM) for 24 h in IAA production medium. Subsequently, HPLC analysis was performed to quantify the IAA production of these strains under different conditions. As anticipated, the EcN-IAA7 strain showed no significant variation in IAA production across the tested tryptophan and thiosulfate concentrations (Fig. 3B). In contrast, the EcN-IAA6 strain exhibited increased IAA titers with higher concentrations of both tryptophan and thiosulfate (Fig. 3A). This correlation between IAA production and the levels of tryptophan and thiosulfate in the EcN-IAA6 strain, while the EcN-IAA7 strain maintained low IAA levels under the same conditions, highlights the responsiveness of the engineered system.

Thiosulfate and nitrate concentrations in the gut are key determinants for the activation of the inflammation-responsive circuits designed in this study. While thiosulfate concentrations in the healthy gut remain poorly characterized, nitrate levels are typically undetectable (< 0.01 mM) under normal conditions but rise to 0.2–0.5 mM during inflammation [44]. The engineered circuits are designed to activate IAA production only within these elevated ranges, ensuring selective response to inflammation markers. This specificity aligns with the objective of minimizing unintended IAA production under healthy gut conditions, as confirmed by the absence of significant production during non-induced experiments.

To ensure stable maintenance of the plasmids encoding IAA biosynthetic genes, we utilized the low-copy-number pBBR1 ori, which has demonstrated superior stability under physiological conditions [45]. This choice minimizes plasmid loss over generations and ensures consistent performance of the genetic circuits. While plasmid retention was not explicitly quantified in this study, prior validation in animal models [28] supports the robustness of this design for long-term therapeutic applications.

Collectively, these findings suggest that the engineered EcN strains with the pPhsA-driven IAA-producing plasmid can effectively detect and respond to varying concentrations of tryptophan and thiosulfate, leading to dose-dependent IAA production for both compounds.

### Development of the nitrate-inducible IAA production

Inflammatory conditions in the gut stimulate the increased expression of nitric oxide synthase (iNOS), leading to higher levels of nitric oxide (NO) production. NO, a reactive nitrogen species, readily converts into nitrate (NO_3_^−^) [46]. Previous research demonstrated the construction of a nitrate-responsive genetic biosensor based on the NarXL two-component regulatory system in the engineered EcN strain, which produces the sfGFP reporter protein in response to nitrate detection. In the presence of nitrate, NarX undergoes phosphorylation, activating NarL. The phosphorylated NarL then activates the expression of the sfGFP reporter gene via the YeaR promoter. These circuits were successfully implemented in a DSS-induced colitis mouse model for non-invasive detection of intestinal inflammation [28]. Building on this success, we aimed to engineer an EcN strain that induces IAA production in response to nitrate, offering an additional therapeutic approach for microbiome-related applications.

**Fig. 4:**
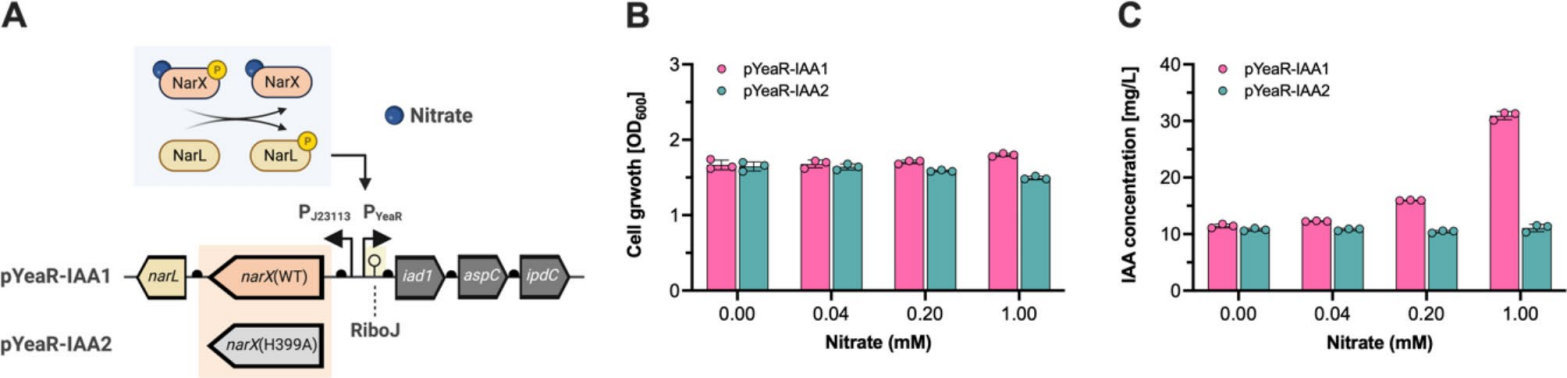
Development and characterization of the nitrate-inducible IAA biosynthesis. (**A**) Schematic representation of the nitrate-inducible IAA-producing plasmids, comprising both the nitrate-responsive genetic circuit and the IAA biosynthesis pathway. The NarX-NarL proteins of the TCS are constitutively expressed from the J23113 promoter. Upon nitrate presence, the constitutively expressed NarX becomes phosphorylated and, in turn, phosphorylates NarL. This phosphorylated NarL then activates the IAA biosynthesis pathway via the YeaR promoter. The pYeaR-IAA1 and pYeaR-IAA2 plasmids contain an operon consisting of the *iad1*, *aspC*, and *ipdC* genes, each with the B0034 RBS, and include a RiboJ insulator upstream of the *iad1* gene. Additionally, the pYeaR-IAA2 plasmid incorporates the NarX(H399A) null mutant. (**B**) Cell growth (OD_600_) and (**C**) IAA titer (mg/L) for pYeaR-IAA1 and pYeaR-IAA2 in the engineered EcN strains with increasing nitrate concentrations (0, 0.04, 0.2, and 1 mM). The engineered EcN strains were cultivated in an IAA production medium with 2 g/L tryptophan and various nitrate levels for 24 h at 37 °C. After that, cell growth and IAA concentrations were measured using a UV-VIS spectrophotometer and HPLC analysis, respectively. Data represent the mean and SD from three biological replicates

To achieve this, we designed genetic circuits comprising the NarXL TCS, which phosphorylates upon binding to nitrate; the PYeaR promoter, activated by phosphorylated NarL; and an IAA production cassette (RiboJ-iad1-aspC-ipdC) under the control of the YeaR promoter. Additionally, we generated a NarX(H339A) null mutant by inactivating the NarX sensory histidine kinase to prevent gene expression under the YeaR promoter, even in the presence of nitrate (Fig. 4A). We transformed EcN cells with two plasmids: pYeaR-IAA1 (NarXL-pYeaR-RiboJ-iad1-aspC-ipdC) and pYeaR-IAA2 (NarX(H399A)L-pYeaR-RiboJ-iad1-aspC-ipdC), resulting in the EcN-IAA8 and EcN-IAA9 strains, respectively.

We then exposed the EcN-IAA8 and EcN-IAA9 strains to varying nitrate concentrations (0 to 1 mM) and measured IAA production. After 24 h in IAA production medium, we observed no significant difference in cell growth across the tested nitrate concentrations for both strains (Fig. 4B). However, the EcN-IAA8 strain exhibited a dose-dependent increase in IAA production with increasing nitrate concentrations. Specifically, the EcN-IAA8 strain produced 30.95 ± 0.73 mg/L of IAA at 1 mM nitrate, whereas the EcN-IAA9 strain produced only 11.07 ± 0.65 mg/L IAA, indicating a 2.8-fold increase in IAA production by the EcN-IAA8 strain (Fig. 4C).

Compared to the thiosulfate-inducible system, the nitrate-inducible system produced lower IAA levels. To further enhance IAA production in the nitrate-inducible system, several strategies can be pursued: (1) incorporating stronger constitutive promoters to enhance *narXL* gene expression; (2) improving nitrate-responsive genetic biosensors for better operational range and induction fold; (3) engineering the EcN chassis to boost L-tryptophan import and its intracellular production using metabolic engineering strategies; and (4) screening or engineering the enzymes Iad1, AspC, and IpdC for more efficient alternatives using IAA-sensing biosensors.

These results demonstrate that the engineered EcN strain with the pYeaR-driven IAA-producing plasmid can detect and respond to varying nitrate concentrations, leading to dose-dependent IAA production. The chronic nature of intestinal inflammation necessitates the design of living therapeutics that integrate multi-input diagnostics and control systems to mitigate prolonged inflammation-related conditions. Our goal is to design genetic circuits responsive to both thiosulfate and nitrate, creating logical gates (AND, OR, NOT, NOR, and NAND) for precise therapeutic intervention. This advancement will pave the way for immune-responsive therapeutics, logically programmed to synthesize IAA in response to gut inflammation.

### Development of IAA biosensors

The limited availability of methods to quantify microbial tryptophan catabolites, particularly IAA, in biological samples underscores the need for improved quantitative analytical techniques that can measure a broader spectrum of these metabolites [31]. Genetically encoded biosensors are valuable tools for high-throughput screening of pathway engineering and strain optimization, enabling the efficient production of value-added compounds. In this context, genetically encoded biosensors are invaluable not only for engineering the IAA biosynthesis pathway but also for optimizing the IAA production process itself.

**Fig. 5:**
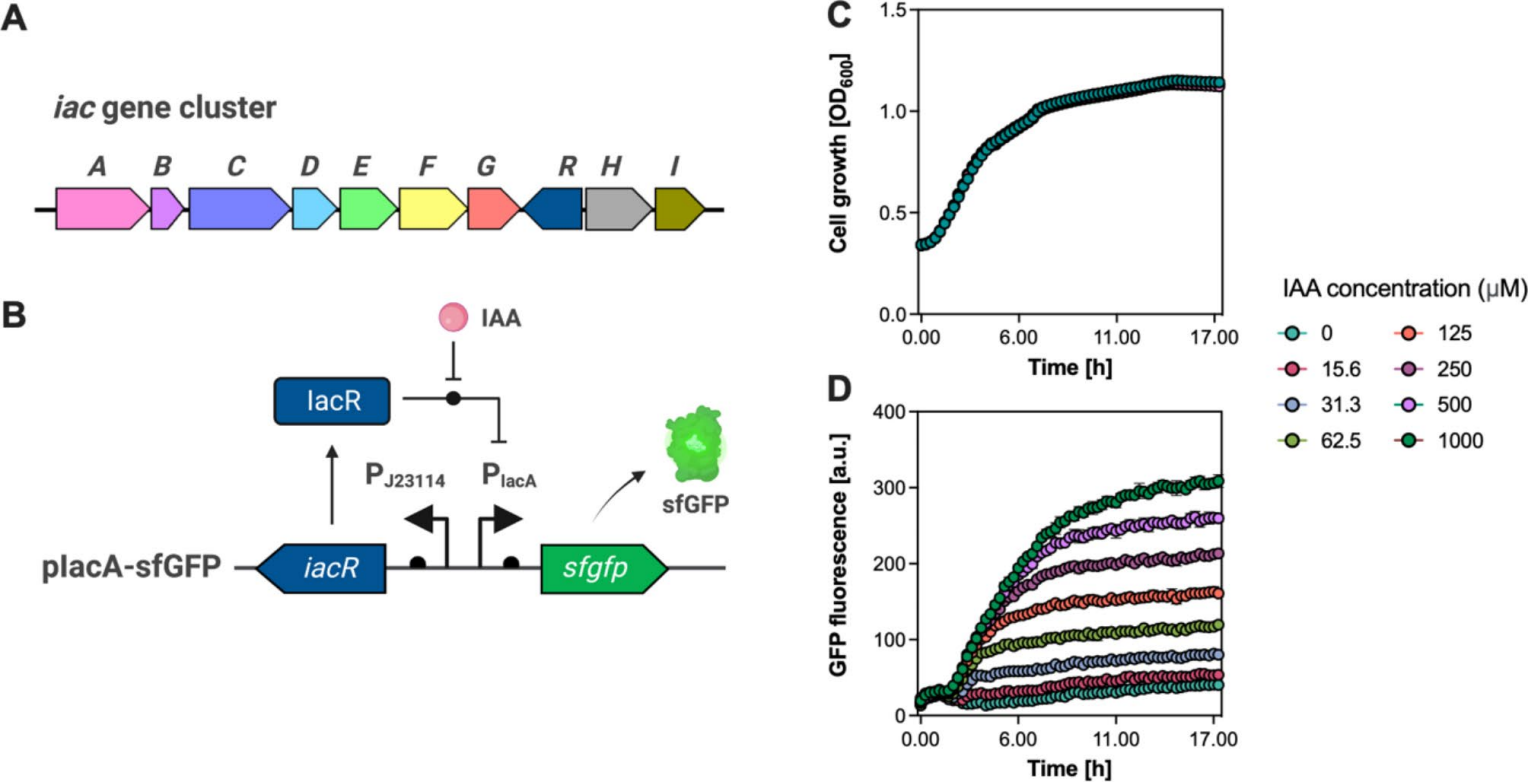
Development and characterization of the IAA-responsive biosensor. (**A**) Organization of the *iacABCDEFGHI* operon and IacR regulator responsible for IAA catabolism in the *P. putida* 1290 strain. (**B**) Schematic representation of the IAA-responsive biosensor in EcN. The IacR regulator from *P. putida* 1290 is continuously expressed under the control of the P_*J23114*_ promoter, while the P_*iacA*_ promoter governs the expression of the *sfgfp* reporter gene. In the absence of IAA, the IacR protein is repressing the P_*iacA*_ promoter; however, in the presence of IAA, IAA binds to the IacR regulator, relieves the repression, and triggers the expression of *sfgfp* reporter gene. (**C and D**) Time-course measurements of (**C**) cell growth and (**D**) fluorescence intensities of the EcN-pIacR-sfGFP strain in response to varying concentrations of IAA (0–1.0 mM) added to the medium. The EcN-pIacR-sfGFP strain was cultivated in the LB medium supplemented with various concentrations of IAA ranging from 0 to 1 mM. Measurements of cell growth (OD_600_) and GFP fluorescent signal (a.u.) were conducted during the 24 h incubation at 37 °C in a Tecan Infinite 200 PRO microplate reader. Data represent the mean and SD from three biological replicates

As a proof of concept, we developed an IAA-biosensor to detect IAA concentrations in the EcN strain. The genes responsible for IAA catabolism were identified in *Pseudomonas putida* 1290 [47, 48]. The indole-3-acetic acid catabolism (*iac*) locus in *P. putida* 1290 includes the catabolism genes *iacABCDEFGHI* and the MarR-family transcriptional regulator IacR (Fig. 5A). The IacR regulator acts as a repressor of the *iac* operon, and transcriptional profiling in *P. putida* 1290 revealed that the *iac* genes are expressed in the presence of IAA, which relieves the repression caused by IacR and is responsible for IAA metabolism.

Leveraging this natural regulatory mechanism, we developed an IAA-biosensor for measuring IAA levels in the EcN strain. We engineered a single plasmid, named placA-sfGFP, derived from the pSEVA131 plasmid, which carries the IacR regulator, the IacA promoter, and the sfGFP reporter gene. The expression of the *iacR* gene is constitutively driven by the J23114 promoter, while the *sfgfp* reporter gene is under the control of the IacR-responsive IacA promoter (Fig. 5B). In the absence of IAA, the IacR protein represses the IacA promoter. However, when IAA is present, it binds to the IacR regulator, alleviating the repression and leading to pIacA-controlled expression of the *sfgfp* reporter gene.

To assess the correlation between exogenously added IAA and the fluorescence output signal, we introduced the placA-sfGFP plasmid into EcN cells, creating the EcN-pIacA-sfGFP strain. We subjected the engineered EcN strain to increasing concentrations of IAA (0 to 1 mM) in LB medium and monitored their growth and fluorescence responses in real time (Fig. 5C and D). We observed no significant difference in cell growth (OD_600_) among the various IAA concentrations tested (Fig. 5C). However, the fluorescence signal from the engineered EcN strain increased in a dose-dependent manner with higher IAA concentrations. Specifically, at an IAA concentration of 1 mM, there was a 7.65-fold increase in fluorescence output compared to the condition with no exogenously added IAA. The IAA-biosensor exhibited a minimum detection concentration of 31.3 µM (Fig. 5D).

While further enhancements are needed to improve the performance of the IAA-biosensor, this represents the first effort, to our knowledge, to construct an IAA-biosensor using the IacR regulator in the EcN strain.

## Conclusion

In this study, we successfully engineered the probiotic strain EcN for targeted production of IAA in response to specific inflammatory biomarkers, namely thiosulfate and nitrate. By optimizing the IAA biosynthetic pathway and integrating the RiboJ insulator, we enhanced IAA synthesis. Additionally, we employed genetic circuits responsive to inflammatory signals to precisely initiate IAA production. Our results demonstrate that the engineered EcN strains can detect and respond to elevated levels of thiosulfate and nitrate, leading to dose-dependent increases in IAA production. Specifically, the strains EcN-IAA4 and EcN-IAA6 showed significant IAA production when exposed to thiosulfate, while the EcN-IAA8 strain exhibited a marked increase in IAA synthesis in response to nitrate. This specificity was confirmed by comparing strains with null mutations in the response regulators, which did not produce IAA in the absence of their respective inducers. Furthermore, we developed an IAA-responsive biosensor using the IacR transcription factor from *P. putida* 1290. This biosensor effectively detected IAA levels, providing a minimum detection concentration of 31.3 µM and showing promise for real-time monitoring of IAA in various biological contexts.

The engineered EcN strains, with their ability to produce IAA in response to inflammatory signals, hold significant potential for the treatment of IBD and similar conditions. The research underscores the feasibility of microbiome engineering to address complex diseases by modulating gut microbiota-derived metabolites. Future work will focus on confirming these findings in murine models of IBD and further enhancing the strains for practical therapeutic applications. In summary, this study advances the field of live biotherapeutic products by demonstrating a novel approach to engineer probiotic strains for inflammation-responsive IAA production, opening new avenues for targeted therapy in gut inflammation and potentially other microbiome-related diseases.

## Electronic supplementary material

Below is the link to the electronic supplementary material.

Supplementary Material 1

## Data Availability

No datasets were generated or analysed during the current study.

## Abbreviations

EcN: *Escherichia coli* Nissle 1917
IAA: Indoleacetic acid
IPyA: Indolepyruvic acid
AHR: Aryl hydrocarbon receptor
IAAId: Indoleacetaldehyde
IPTG: Isopropyl-β-D-thiogalactopyranoside
LB: Lysogeny broth
OD: Optical density
sfGFP: Superfolder green fluorescent protein
HPLC: High-performance liquid chromatography
RID: Refractive index detector
UV-VIS: Ultraviolet-visible
TCS: Two-component system
IL-22: Interleukin-22
IBD: Inflammatory bowel disease
DSS: Dextran sodium sulfate
LBP: Live biotherapeutic product
RBS: Ribosome binding site
CD: Crohn’s disease
UC: Ulcerative colitis
