# Supplementary material for: Engineering probiotic Escherichia coli for inflammation-responsive indoleacetic acid production using RiboJ-enhanced genetic circuits
Source: J Biol Eng. 2025 Jan 21;19:10. doi: 10.1186/s13036-025-00479-y (PMC11753152; doi:10.1186/s13036-025-00479-y)
Supplement: Supplementary file 1 — Supplementary Material 1 [file 13036_2025_479_MOESM1_ESM.pdf]

SUPPLEMENTARY INFORMATION

Engineering Probiotic *Escherichia coli* for Inflammation-Responsive Indoleacetic Acid Production Using RiboJ-Enhanced Genetic Circuits

Seung-Gyun Woo[1,2], Seong Keun Kim[1], Seung-Goo Lee[1,3,4]* and Dae-Hee Lee[1,3,4,5]*

[1]Synthetic Biology Research Center, Korea Research Institute of Bioscience and Biotechnology (KRIBB), Daejeon 34141, Republic of Korea
[2]Department of Molecular Biosciences, University of Texas at Austin, Austin, Texas 78712, USA
[3]Department of Biosystems and Bioengineering, KRIBB School of Biotechnology, University of Science and Technology (UST), Daejeon 34113, Republic of Korea
[4]Graduate School of Engineering Biology, Korea Advanced Institute of Science and Technology (KAIST), Daejeon 34141, Republic of Korea
[5]Department of Integrative Biotechnology, College of Biotechnology and Bioengineering, Sungkyunkwan University, Suwon-si, Gyeonggi-do 16419, Republic of Korea

***Corresponding authors.**
Dae-Hee Lee, Ph.D., Tel: +82 42 879 8225; E-mail: dhlee@kribb.re.kr
Seung-Goo Lee, Ph.D., Tel: +82 42 860 4373; E-mail: sglee@kribb.re.kr

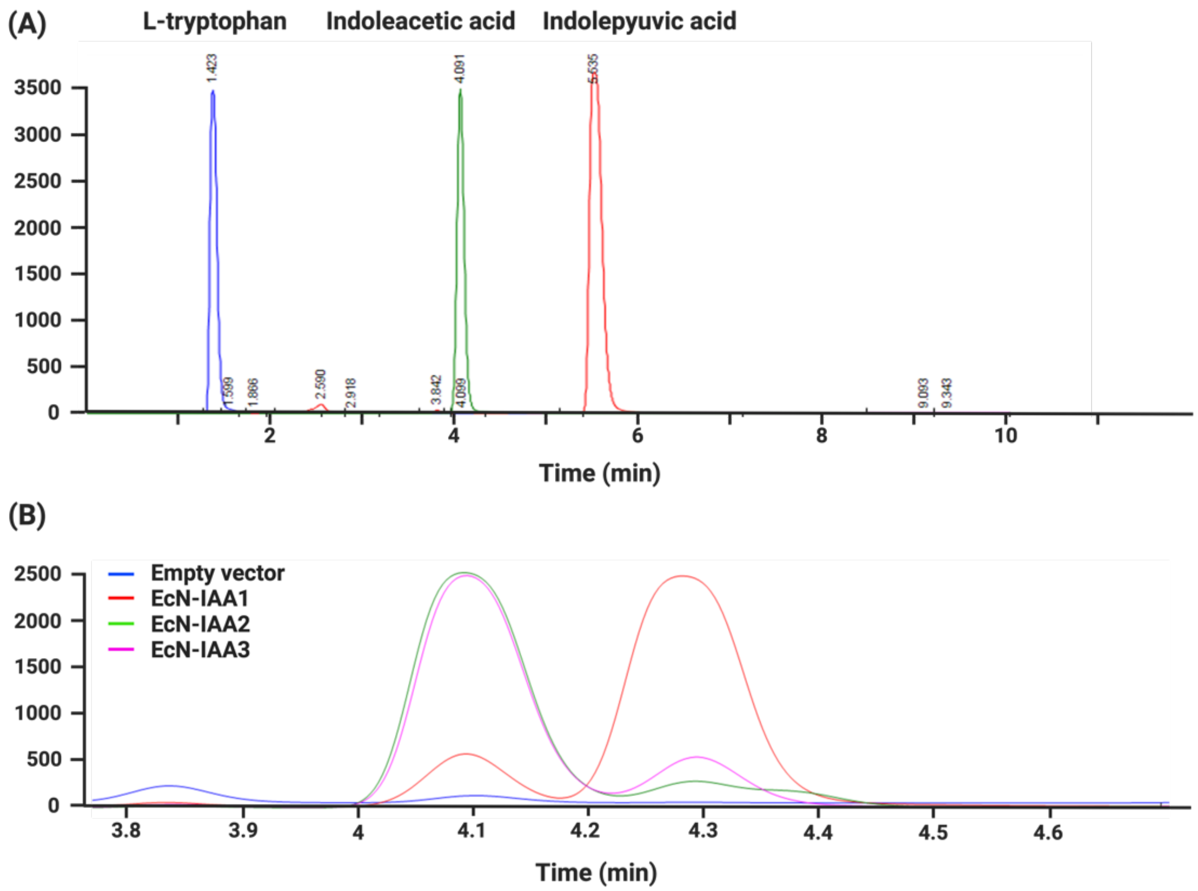

Figure S1. Representative HPLC chromatograms of both standard substances and analytes. All analytes are Identified, along with their retention times, as shown in panels A and B. Panel A represents the standard mixture, while Panel B illustrates the analyte samples.

Table S1. Bacterial strains and plasmids used in this study.

Name	Description	Reference
E. coli strains		
DH5α	*F–, Φ80lacZ·ΔM15·f(lacZYA-argF)U169 deoR recA1 endA1 hsdR17(rk,-mk+) phoA supE44 thi-1 gyrA96 relA1*	Enzynomics
Nissle 1917 (EcN)	Wild-type strain (serotype O6:K5:H1)	GmbH
Plasmids		
pTrc99A	P_{trc} promoter, $lacI^{q}$, pBR322 ori, Amp^{R}	GE Healthcare
pTSN-IAA1	pTrc99A derivatives containing *aspC* of *E. coli*, codon-optimized *ipdC* of *Enterobacter cloacae*, and codon-optimized *iad1* of *Ustilago maydis*, pBR322 ori, Amp^{R}	This study
pTSN-IAA2	pTrc99A derivatives containing iad1-aspC-ipdC gene cluster, pBR322 ori, Amp^{R}	This study
pTSN-IAA3	pTrc99A derivatives containing RiboJ-iad1-aspC-ipdC gene cluster, pBR322 ori, Amp^{R}	This study
pSEVA131-J23114-TPG	Derivative of pSEVA131 harboring *thsS*(L547T)*R* genes, constitutive promoter (J23114), output promoter P_{phsA}, and *sfgfp* gene, pBBR1 ori, Amp^{R}	(1)
pSEVA131-J23114-T(D57A)PG	Derivative of pSEVA131 harboring *thsS*(L547T)*R*(D57A) genes, constitutive promoter (J23114), output promoter P_{phsA}, and *sfgfp* gene, pBBR1 ori, Amp^{R}	(1)
pPhsA-IAA4	Derivative of pSEVA131 harboring *thsS*(L547T)*R* genes, constitutive promoter (J23114), output promoter P_{phsA}, and iad1-aspC-ipdC gene cluster, pBBR1 ori, Amp^{R}	This study
pPhsA-IAA5	Derivative of pSEVA131 harboring *thsS*(L547T)*R*(D57A) genes, constitutive promoter (J23114), output promoter P_{phsA}, and iad1-aspC-ipdC gene cluster, pBBR1 ori, Amp^{R}	This study
pPhsA-IAA6	Derivative of pSEVA131 harboring *thsS*(L547T)*R* genes, constitutive promoter (J23114), output promoter P_{phsA}, and RiboJ-iad1-aspC-ipdC gene cluster, pBBR1 ori, Amp^{R}	This study
pPhsA-IAA7	Derivative of pSEVA131 harboring *thsS*(L547T)*R*(D57A) genes, constitutive promoter (J23114), output promoter P_{phsA}, and RiboJ-iad1-aspC-ipdC gene cluster, pBBR1 ori, Amp^{R}	This study
pSEVA131-J23113-NPG	Derivative of pSEVA131 harboring *narXL* genes, constitutive promoter (J23113), output promoter P_{yeaR}, and *sfgfp* gene, pBBR1 ori, Amp^{R}	(1)
pSEVA131-J23113-N(H399A)PG	Derivative of pSEVA131 harboring *narX*(H399A)*L* genes, constitutive promoter (J23113), output promoter P_{yeaR}, and *sfgfp* gene, pBBR1 ori, Amp^{R}	(1)
pYeaR-IAA1	Derivative of pSEVA131 harboring *narXL* genes, constitutive promoter (J23113), output promoter P_{yeaR}, and RiboJ-iad1-aspC-ipdC gene cluster, pBBR1 ori, Amp^{R}	This study
pYeaR-IAA2	Derivative of pSEVA131 harboring *narX*(H399A)*L* genes, constitutive promoter (J23113), output promoter P_{yeaR}, and RiboJ-iad1-aspC-ipdC gene cluster, pBBR1 ori, Amp^{R}	This study
pIacA-sfGFP	Derivative of pSEVA131 harboring codon-optimized *iacR* of *Pseudomonas putida*, constitutive promoter (J23114), output promoter P_{iacA}, and *sfgfp* gene, pBBR1 ori, Amp^{R}	This study

Table S2. DNA oligonucleotides used in this study.

Name	Sequence (5' to 3')	Purpose
aspC-IF	ttcacacaggaggttaaaccatgtttgagaacattaccgc	For the construction of the pTSN-IAA1 plasmid
aspC-IR	tttctcctctttgcggccgcttacagcactgccacaatcg	
aspC-VF	gcggccgcaaagaggagaaatactagatgcgtaccccgta	
iad1-IR	cttgcatgcctgcaggtcgatcacgctttcgggatccaca	
iad1-VF	tgtggatcccgaaagcgtgatcgacctgcaggcatgcaag	
aspC-VR	gcggtaatgttctcaaacatggtttaacctcctgtgtgaa	
iad1-IF	ttcacacaggaggttaaaccatgaccgttggtgttgcgtc	For the construction of the pTSN-IAA2 plasmid
iad1-IR	ctagtatttctcctctttgcggccgctcacgctttcgggatccaca	
aspC(B0034)-IF	gcggccgcaaagaggagaaatactagatgtttgagaacattaccg	
ipdC-IR	cttgcatgcctgcaggtcgatcacgcgttgttacgcgctt	
rrnBT-VF	tcgacctgcaggcatgcaagcttggctgttttggcggatg	
lacO-VR	ggtttaacctcctgtgtgaaattgttatccgctcacaatt	
RiboJ-IF	agcggataacaattagctgtcaccggatgtgctt	For the construction of the pTSN-IAA3 plasmid
RiboJ-IR	aacctcctgtgtgattaaacaaaattatttgtag	
RiboJ(iad1)-VF	gtttaatcacacaggaggttaaaccatgaccgtt	
ipdC-IR	cttgcatgcctgcaggtcgatcacgcgttgttacgcgctt	
rrnBT-VF	tcgacctgcaggcatgcaagcttggctgttttggcggatg	
RiboJ(lacO)-VR	acagctaattgttatccgctcacaattccacaca	
iad1(B0034)-IF	aaagaggagaaatactagatgaccgttggtgttg	For the construction the of pPhsA-IAA1 and pPhsA-IAA2 plasmids
ipdC-IR1	aggatctcacgcgttgttacgcgcttccagcgct	
ipdC-VF	gtaacaacgcgtgagatcctacaaggaggtacca	
ThsS(B0034)-VR	atctagtatttctcctctttgttaaatctcccaatgacag	
RiboJ(PphsA)-IF	tttaacagctgtcaccggatgtgctttccggtct	For the construction the of pPhsA-IAA3 and pPhsA-IAA4 plasmids
ipdC-IR1	aggatctcacgcgttgttacgcgcttccagcgct	
ipdC-VF	gtaacaacgcgtgagatcctacaaggaggtacca	
RiboJ(PphsA)-VR	atccggtgacagctgttaaatctcccaatgacag	
RiboJ(PyeaR)-IF	aaaaggagctgtcaccggatgtgctttccggtct	For the construction the of pYeaR-IAA1 and pYeaR-IAA2 plasmids
ipdC-IR1	aggatctcacgcgttgttacgcgcttccagcgct	
ipdC-VF	gtaacaacgcgtgagatcctacaaggaggtacca	
RiboJ(PyeaR)-VR	atccggtgacagctccttttccctccttcggtga	
iacR-IF	acacaggaaagcttttacagggtttccaggttgt	For the construction of the placA-sfGFP plasmid
PiacA(B0034)-IR	tatttctcctctttggacattctccgtctcaggc	
PiacA(B0034)-VF	atgtccaaagaggagaaatactagatgagcaaag	
iacR-VR	ctgtaaaagctttcctgtgtgaaattgttatccg	

Table S3. DNA sequences of *iad1*, *aspC*, *ipdC,* and *iacR* used in the study.

>iad1

atgaccgttggtgttgcgtctaaactgctggacgcgccgttctctcacgttatcaacggtaaatctgttgacacccagggtttcaaaaccctggacg
ttatcgacccggcgaccgaaaaagcgatcgcgcaggttccgatcgcgaccaaagaaaccgttgaccaggcggttgacgcggcgcacgcggcg
ttcccgtcttggtctaaaaccacctgggacgaacgtgcgaaactgctggaacagtacggtgaagaatacaaagcgatgctgccggaactggtta
aactgctgaccgcggaacagggtaaatctatccagttcgcgcagcacgaatgcgaaaccatcctgccgtggttcaccgaactggcgaaagttcg
tctggacgaaaaagttgttcacgaaaacgacacccacaaagcgatcgaacgttacgttccgctgggtgtttgcgcgggtatcgttccgtggaactt
cccggttctgctgatgctgtggaaagttacccaggcgatcgttaccggtaactgcatcatcatcaaaccgtctccgttcaccccgctgtgcgacat
ccgtatcatcgaagcggcgcagaaagttttcccgccgggtgttgttcagatcgttgttggtgacgacaacctgggtccgtggatcaccgaacaccc
gcgtatccagaaaatctctttcaccggttctaccgcgaccggtaaactggttgcgaaatcttgctctgcgaccctgaaacgtttcaccctggaact
gggtggtaacgacccgctggttgttctgccgcagcagaaagacctggcggcgaccgcgcagaacgttctgctggcggcgttcttcaactctggtc
aggtttgcatcgcggcgaaacgtatctacatccacgaagacatctacgacgaattcaaagcgaccctggcgcaggttgttcagcacttcaaagtt
ggtccgggtaacgaagaaggtgttatgttcggtccgatcaacaacaaaatgcagtacaacaaagttggtgaattcttcgaagaagcgaaaaaaa
acaacttcaacctggttgcgggtggtcaggttgaagaaaaaccgggtttcttctacccgctgaccatcgttgacaacccgccggaaaactctcgt
ctggttcaggaagaaccgttcggtccgatcgttccgctgctgaaatggaaagacgaagcggacctggttaaacgtatcaacgactctcagtgggg
tctgggtgcgtctgtttggtcttctgacatccaggcggcggaacgtatcggtcgtcagatcgaatctggtaccatctggatcaactgcctggttcacc
accacccggcggttccgttcggtggtttcaaatcttctggtatcggtgcggaacacggtaaaaacggtctgggtgcgtactgccaggttcaggcgct
gtggatcccgaaagcgtga

>aspC

atgtttgagaacattaccgccgctcctgccgacccgattctgggcctggccgatctgtttcgtgccgatgaacgtcccggcaaaattaacctcggg
attggtgtctataaagatgagacgggcaaaaccccggtactgaccagcgtgaaaaaggctgaacagtatctgctcgaaaatgaaaccaccaaa
aattacctcggcattgacggcatccctgaatttggtcgctgcactcaggaactgctgtttggtaaaggtagcgccctgatcaatgacaaacgtgctc
gcacggcacagactccgggggcactggcgcactacgcgtggctgccgatttcctggcaaaaaataccagcgttaagcgtgtgtgggtgagcaa
cccaagctggccgaaccataagagcgtctttaactctgcaggtctggaagttcgtgaatacgcttattatgatgcggaaaatcacactcttgacttc
gatgcactgattaacagcctgaatgaagctcaggctggcgacgtagtgctgttccatggctgctgccataacccaaccggtatcgaccctacgct
ggaacaatggcaaacactggcacaactctccgttgagaaaggctggttaccgctgtttgacttcgcttaccagggttttgcccgtggtctggaaga
agatgctgaaggactgcgcgctttcgcggctatgcataaagagctgattgttgccagttcctactctaaaaactttggcctgtacaacgagcgtgttg
gcgcttgtactctggttgctgccgacagtgaaaccgttgatcgcgcattcagccaaatgaaagcggcgattcgcgctaactactctaacccacca
gcacacggcgcttctgttgttgccaccatcctgagcaacgatgcgttacgtgcgatttgggaacaagagctgactgatatgcgccagcgtattcag
cgtatgcgtcagttgttcgtcaatacgctgcaggaaaaaggcgcaaaccgcgacttcagctttatcatcaaacagaacggcatgttctccttcagt
ggcctgacaaaagaacaagtgctgcgtctgcgcgaagagtttggcgtatatgcggttgcttctggtcgcgtaaatgtggccgggatgacaccagat
aacatggctccgctgtgcgaagcgattgtggcagtgctgtaa

>ipdC

atgcgtaccccgtactgcgttgcggactacctgctggaccgtctgaccgactgcggtgcggaccacctgttcggtgttccgggtgactacaacctg
cagttcctggaccacgttatcgactctccggacatctgctgggttggttgcgcgaacgaactgaacgcgtcttacgcggcggacggttacgcgcgt
tgcaaaggtttcgcggcgctgctgaccaccttcggtgttggtgaactgtctgcgatgaacggtgttgcgggttctttcgcggaacacgttccggttctg

cacatcgttggtgcgccgggtatggcggcgcagcagcgtggtgaactgctgcaccacaccctgggtgacggtgaattccgtcacttctaccacat
gtctgaaccgatcaccgttgcgcaggcggttctgaccgaacagaacgcgtgctacgaaatcgaccgtgttctgaccaccatgctgcgtgaacgt
cgtccgggttacctgatgctgccggcggacgttgcgaaaaaagcggcgaccccgccggtttctgcgctgaccgttaacccggcgccggcggac
ctggcgtgcctgcaggcgttccgtgaagcggcggaaaaacgtctgtctacctctaaacgtaccgcgctgctggcggacttcctggttctgcgtcac
ggtctgcgtaccgcgctgcagacctgggttaaagaagttccgatggcgcacgcgaccatgctgatgggtaaaggtatcttcgacgaacgtcagtct
ggtttctacggtacctactctggttctgcgtctgcggcgccggttaaagaagcgatcgaaggtgcggacaccgttctgtgcatcggtacccgtttca
ccgacaccctgaccgcgggtttcacccaccagctgaccccggaccagaccatcgaagttcagccgcacgcgtctcgtgttggtgacgtttggtt
caccggtatcccgatgcgtgaagcgatcgaaaccctgaccgcgctgtgcaaaacctacgttcgtgacacccgtgcgccgctggaccactctggt
ttctctttcccgaccatcgaaggtgcgctgacccaggaatctttctggcgtaccctgcagaccttcatccgtccgggtgacatcatcctggcggac
cagggtacctctgcgttcggtgcgatcgacctgcgtctgccggcggacgttaacttcatcgttcagccgctgtggggttctatcggttacaccctggc
ggcggcgttcggtgcgcagaccgcgtgcccgaaccgtcgtgttatcgttctgaccggtgacggtgcggcgcagctgaccatccaggaactgggtt
ctatgctgcgtgacaaacagcgtccgatcatcctggttctgaacaacgaaggttacaccgttgaacgtgcgatccacggtccggaacagcgttac
aacgacatcgcgctgtggaactggacccagatcccgcaggcgctgtctctggcgccgcaggcggaatgctggcgtgtttctgaagcggaagcgc
tggcggaagttctggacaaagttgcgcaccacgaacgtctgtctctgatcgaagttatgctgccgaaagcggacatcccgccgctgctgtctgcg
ctgaccaaagcgctggaagcgcgtaacaacgcgtga

>iacR

atgtctaacgcgaaaaacacctctgcggcgtctccggcgcgtaaaggtcactctcaccacgacccggcgtctgacgaattccgtaaagaagac
ttcccgttctactggctggcgcgtgttcacggtcgttacacccagaacatggaacgtctgctgaaaaaaatcgacctggacgttccgcgttggcgt
gttctgtggatcctgaacgaaaacggtgaatcttctatctctgaaatctctacccacgcgatcgcgaaactgtctaccatcaccaaaatcgtttac
cgtatgaaagaagacggtctggttgacaccgcgccgtctccggaagacggtcgtgttacccaggttcgtatcaccgaagttggtctgcagaacat
cgaacgtatgcaggaagttacccgtgaactgttccagcgttctttcaaaggtctgaccgaagcgcaggttcagcgtctgaaccgtatgctggaagt
tgttttccacaacctggaaaccctgtaa

References

1. Woo, S.G., Moon, S.J., Kim, S.K., Kim, T.H., Lim, H.S., Yeon, G.H., Sung, B.H., Lee, C.H., Lee, S.G., Hwang, J.H. and Lee, D.H. (2020) A designed whole-cell biosensor for live diagnosis of gut inflammation through nitrate sensing. *Biosens Bioelectron*, **168**, 112523.
